# Long-range Stripe Nanodomains in Epitaxial (110) BiFeO_3_ Thin Films on (100) NdGaO_3_ Substrate

**DOI:** 10.1038/s41598-017-05055-z

**Published:** 2017-07-07

**Authors:** Yogesh Sharma, Radhe Agarwal, Charudatta Phatak, Bumsoo Kim, Seokwoo Jeon, Ram S. Katiyar, Seungbum Hong

**Affiliations:** 1 Materials Science Division, Argonne National Laboratory, Lemont, IL 60439 USA; 2 Department of Physics and Institute for Functional Nanomaterials, University of Puerto Rico, San Juan, PR 00936 USA; 3 Department of Materials Science and Engineering, KAIST, Daejeon, 34141 Korea; 4 Present Address: Materials Science and Technology Division, Oak Ridge National Laboratory, Oak Ridge, TN 37831 USA

## Abstract

Here, we report the observation of ferroelectric and ferroelastic nanodomains in (110)-oriented BiFeO_3_ (BFO) thin films epitaxially grown on low symmetric (100) NdGaO_3_ (NGO) substrate. We observed long range ordering of ferroelectric 109° stripe nanodomains separated by periodic vertical domain walls in as-grown 130 nm thick BFO films. The effect of La_0.67_Sr_0.33_CoO_3_ (LSCO) conducting interlayer on domain configurations in BFO/NGO film was also observed with relatively short range-ordering of stripe domains due to the modified electrostatic boundary conditions in BFO/LSCO/NGO film. Additional studies on B-site doping of Nb ions in BFO films showed change in the domain structures due to doping induced change in lattice anisotropy while maintaining the stripe domain morphology with 109° domain wall. This long-range array of ferroelectric and ferroelastic domains can be useful for optoelectronic devices and ferroelastic templates for strain coupled artificial magnetoelectric heterostructures.

## Introduction

Multiferroics show the simultaneous ordering of charge, spin and lattice parameters, which lead to the spontaneous emergence of physical properties, such as polarization, magnetization, and strain in a single phase of material system^1–4^. These properties can be tuned by external stimuli, such as electric field for ferroelectric materials, magnetic field for ferromagnetic materials, and mechanical stress or strain for ferroelastic materials^2–4^. In ferroelectric and ferroelastic materials, domain formation takes place due to a phase transition to a lower symmetry phase while cooling down below the Curie temperature. In the case of ferroelectric thin films, the size and shape of the domains are determined by the energy competition between electrostatic, strain and domain wall energies. The two main factors that govern this energy competition are presence of screening charges and the thickness of the films^5,6^. In contrast, formation of the ferroelastic domains results from the balance of the elastic energy density against the domain wall energy^7–10^. Therefore, the elastic boundary conditions, such as the epitaxial strain and film thickness determine the size of ferroelastic domains.

The perovskite BiFeO_3_ (BFO) is one of the most widely studied room temperature magnetoelectric multiferroic due to large polarization, high Curie temperature, and relatively low band gap, which makes it an attractive candidate for various applications^11–19^. The magnetoelectric coupling in BFO allows for control of the ferroelectric and magnetic domains via applied electric fields. In epitaxially grown BFO thin films, ferroelectric domain formation can be manipulated by the growth conditions and strain engineering. This epitaxial strain stabilizes the formation of ferroelastically twinned ferroelectric domains, which can be further modified by the change in the substrate-induced strain^16,17^. In such BFO films, ferroelastic domains play an important role in facilitating the coupling between the polarization and the magnetization via the ferroelastic switching of (111) antiferromagnetic plane^15,16^. Direct control over magnetization by electric field via ferroelastic switching potentially facilitates ultralow power voltage controlled spintronics and non-volatile magnetoelectric memory devices^15,17,20^. Therefore, the studies on effects of both compressive and tensile epitaxial strain on the ferroelastic and ferroelectric domain patterns in BFO under different boundary conditions have attracted a great deal of attention.

Recent studies have shown that the (100)/(001) oriented epitaxial rhombohedral BFO films can exhibit complex stripe domain patterns by reducing the substrate symmetry either by choosing high miscut angle cubic substrates (mainly STO (001))^16,21^ or by the use of low symmetry orthorhombic substrates (mainly (110)-cut rare-earth scandate substrates)^17^. Most of the reports are based on the piezoresponse force microscopy (PFM) studies of ferroelastic domains in (100) epitaxial BFO films. Based on the PFM images, stripe domain pattern and domain wall orientation were directly interpreted by the periodic change in phase contrast across domain walls while neglecting the corresponding amplitude contrasts, where amplitude response could also be important for analyzing the domain variants more accurately. In case of (100) oriented films, PFM phase contrast might be sufficient to certain extent, but the interpretation based on only PFM phase contrast could be misleading in case of (110) oriented films, where 4 out of 8 polarization variants lie in the plane of the film^7^. Apart from that, there are not many studies in literature on stripe domain configurations in (110)-oriented BFO films grown on low symmetry substrates^17^. Furthermore, the studies on manipulation of ferroelastic stripe nanodomains in BFO films by; (i) the effect of bottom conducting layer (charge screening) and (ii) change in lattice anisotropy due to cation doping (A or B- site doping), have not been much explored.

In this paper, we report on the ferroelectric and ferroelastic nanodomains formation in 130 nm thick (110) BiFeO_3_ (BFO) thin films epitaxially grown on low symmetric GdFeO_3_-type (100) NdGaO_3_ (NGO) substrate. We used piezoresponse force microscopy (PFM) and charge gradient microscopy (CGM) to observe long range ordering of ferroelectric 109° stripe nanodomains separated by periodic vertical domain walls in as grown BFO films. We also studied the effect of doping and charge screening on stripe domain structure in epitaxial BFO films.

## Results and Discussion

**Figure 1:**
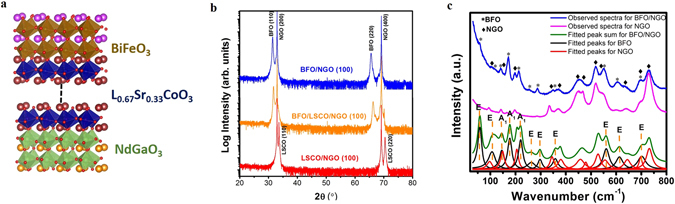
(**a**) Unit cell representation of BFO/LSCO/NGO heterostructure. (**b**) XRD θ‒2θ scan for LSCO/NGO, BFO/LSCO/NGO, and BFO/NGO heterostructures, respectively. (**c**) Observed and fitted Raman spectra of NGO (100) single crystal substrate and 130 nm (110) BFO film grown on (100) NGO.

In Fig. 1b, θ−2θ XRD patterns of La_0.67_Sr_0.33_CoO_3_ (LSCO)/NdGaO_3_ (NGO) (100)_O_ (where O stands for orthorhombic structure), BiFeO_3_ (BFO)/LSCO/NGO, and BFO/NGO heterostructures are presented where BFO film show only hh0 Bragg’s peaks except the orthorhombic NGO (100) and LSCO peaks, implying that BFO film is epitaxially grown in [110] direction. We can correlate orthorhombic unit cell with pseudocubic one, where the [001]_O_, [100]_O_, and [010]_O_ orthorhombic directions correspond to the pseudocubic directions [001], [1$\bar{1}$0], and [110], respectively^17^. Therefore, the NGO (100)_O_ substrate behaves like a (110)-oriented cubic perovskite substrate, which prefers the epitaxial growth of (110)-oriented BFO films. As such, BFO film directly grown on NGO (100)_O_ was epitaxially oriented along [110] direction without secondary phases.

Furthermore, the pseudocubic lattice parameters of LSCO are closely matched with those of NGO, which make LSCO an appropriate perovskite conducting template for BFO films. It has been reported that the large structural anisotropy with the in-plane compressive strain imposed by orthorhombic substrate facilitates the growth of stripe domain structure in BFO film^17^. The vibrational spectra of BFO/NGO heterostructure have also been characterized by Raman spectroscopy. In rhombohedral BFO with R3c space group, a primitive cell contains two BFO formula units which results in 27 zone-center optical phonon modes: 4A1 + 5A2 + 9E (where 9E are doubly degenerate)^22^. Out of these modes, 13 modes, 4A1 and 9E, are Raman active. Figure 1c shows the room temperature Raman spectra of BFO/NGO film and NGO substrate. After fitting Raman spectra using a theoretically obtained Raman line shape (damped harmonic oscillator model)^22^, we observed 11 Raman active modes in BFO/NGO films corresponding to rhombohedral (R3c) symmetry of BFO.

**Figure 2:**
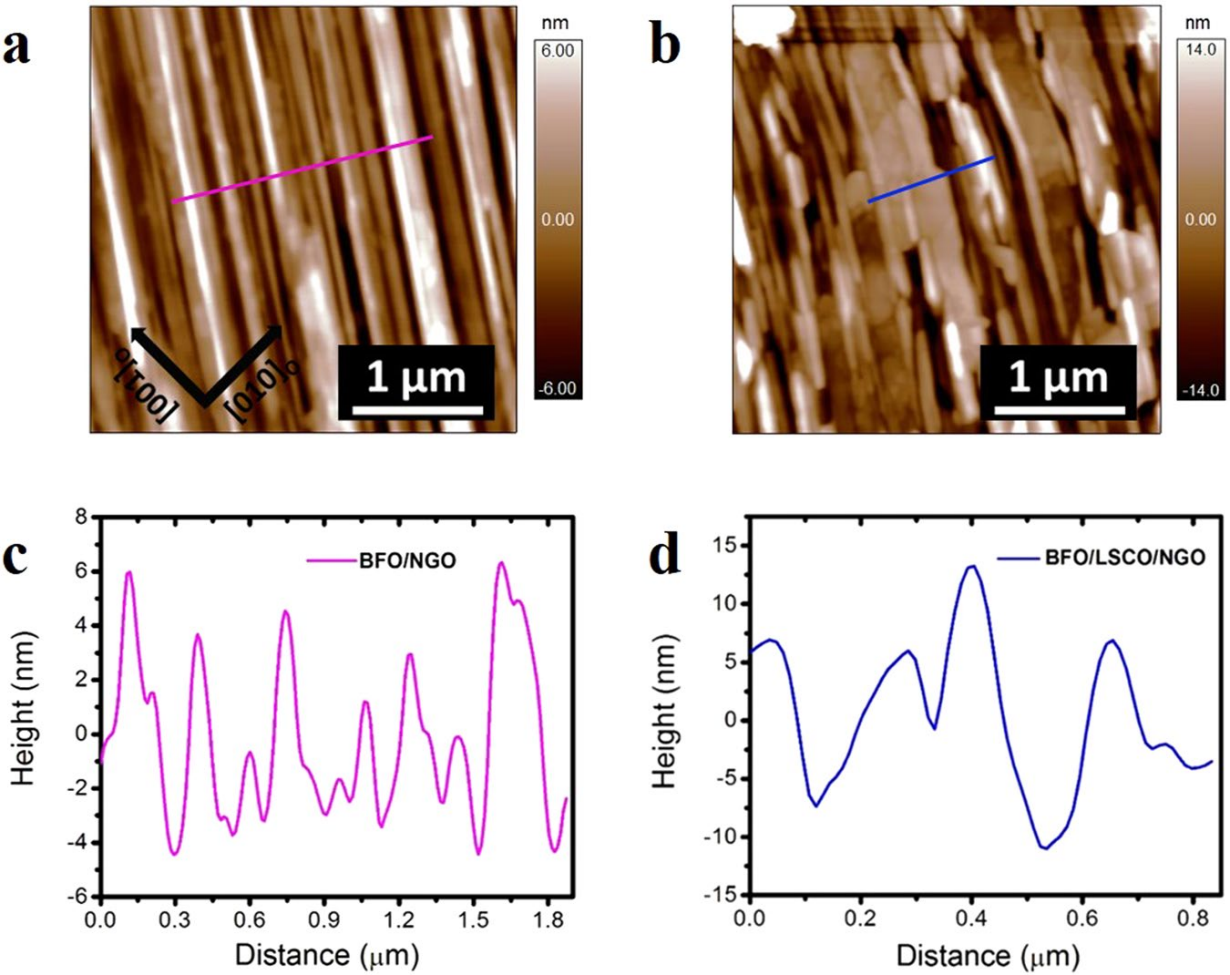
AFM topography images of; (**a**) 130 nm BFO film grown on NGO and (**b**) on 100 nm LSCO layered NGO. (**c**,**d**) Section analysis along the lines drawn in (**c**) and (**d**), respectively, showing the puckering of the surfaces.

Surface morphologies of the BFO films grown on NGO with and without LSCO conducting interlayer were observed by atomic force microscopy (AFM). AFM topography images of BFO/NGO (Fig. 2a) and BFO/LSCO/NGO (Fig. 2b) films show “puckered” surface with root-mean square roughness of ~3.5 nm and 4.2 nm, respectively (over 3 × 3 µm^2^ area). Figure 2c,d depict the section analysis of Fig. 2a,b representing line profiles of the height for both films where the surface puckering angles are estimated to be ~174° and 174.8° [see Fig. S2 for more measurement details]. We observed well-aligned long-range stripe morphology on the surface of BFO/NGO thin films. However, in case of BFO/LSCO/NGO thin films, we observed relatively short-range ordering of stripes.

**Figure 3:**
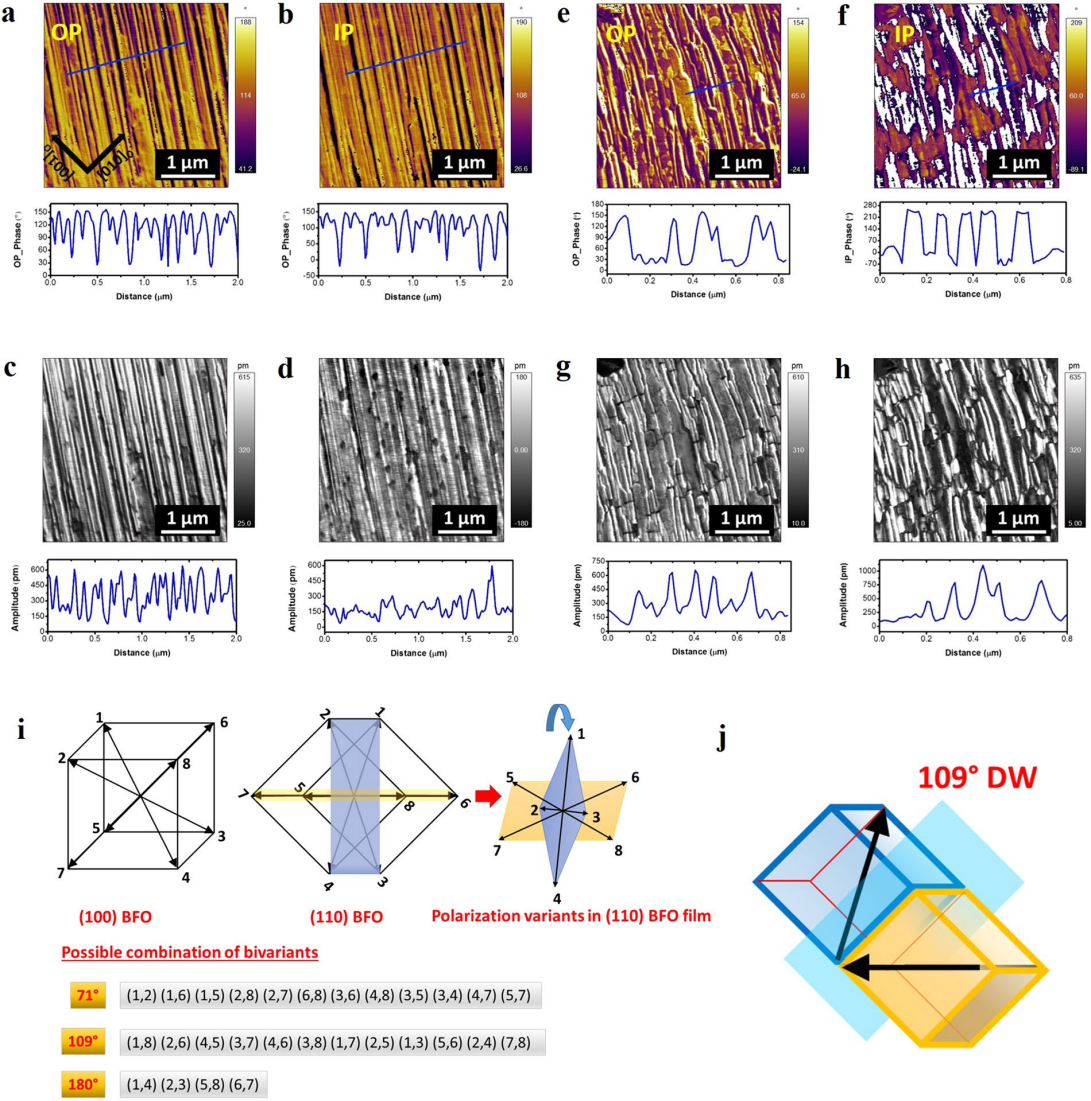
PFM images of BFO films showing out-of-plane (OP) and In-plane (IP) phase and amplitude contrast corresponding to BFO/NGO (**a**–**d**) and BFO/LSCO/NGO (**e**–**h**) heterostructures with stripe nanodomains in the areas of Fig. 2a,b, respectively. (**i**) Schematics of the polarization variants in (100) and (110) BFO films with the possible combinations of bidomain variants corresponding to 71°, 109°, and 180° domain wall orientations. The arrows represent the spontaneous polarization directions. (**j**) Schematic illustration of the 109° domain structure in BFO/NGO heterostructure.

Piezoresponse force microscopy (PFM) images also confirmed the different polarization domain structures in BFO/NGO and BFO/LSCO/NGO thin films presented in Fig. 3a–h. Rhombohedral BFO shows eight polarization variants along <111> direction, where half of them are related by an inversion symmetry^7^. Schematic illustrations showing the eight directions of the spontaneous polarizations for (100) and (110) oriented BFO films are presented in Fig. 3i. Figure 3a–h shows the PFM images of BFO/NGO and BFO/LSCO/NGO thin films scanned in the same areas of Fig. 2a,b, respectively. We observed 109° stripe domain configurations in BFO/NGO film as confirmed from out-of-plane (OP) and in-plane (IP) PFM images (Fig. 3a,b) and corresponding PFM amplitude responses (Fig. 3c,d). The schematic of 109° domain wall orientation is shown in Fig. 3j. The domain configurations in BFO/NGO films were further confirmed by angle resolved PFM measurements (Fig. S3), whereby the sample was rotated by 45° increments around the normal to the film surface, and OP and IP PFM phase and amplitude signals were collected at 0°, 45°, and 90°, respectively^23^. Furthermore, to confirm PFM observations and the domain structure, cross-sectional transmission electron microscopy (TEM) studies were performed on BFO/NGO sample. The appearance of triangular nanodomain^14^ in TEM cross-sectional image further confirms the presence of ferroelectric/ferroeleastic domain walls in our BFO films and the selected area electron diffraction (SAED) pattern from an area of the film including multiple domains also confirms the (110) growth of BFO film (Fig. S4).

In case of BFO/LSCO/NGO film, we observed different domain configurations as shown in Fig. 3e,f. As can be seen from PFM phase and amplitude images, the in-plane component of polarization became wider, and short-range ordering of stripes was observed in BFO films with LSCO interlayer. In case of (100)/(001) epitaxial BFO films, it has been reported that the conducting bottom layer screens the bound surface charges at BFO/substrate interface, which suppresses the depolarization field and thus stabilizes stripe domains with 71° domain wall^14,17^. However, in our (110) oriented BFO films, we observed expansion in the width of in-plane domain variants and shorter stripe domains instead of formation of 71° domain wall, as observed from the PFM phase and amplitude images and possible domain variants schematics outcomes based on the measurement conditions (Figs S5 and S6).

**Figure 4:**
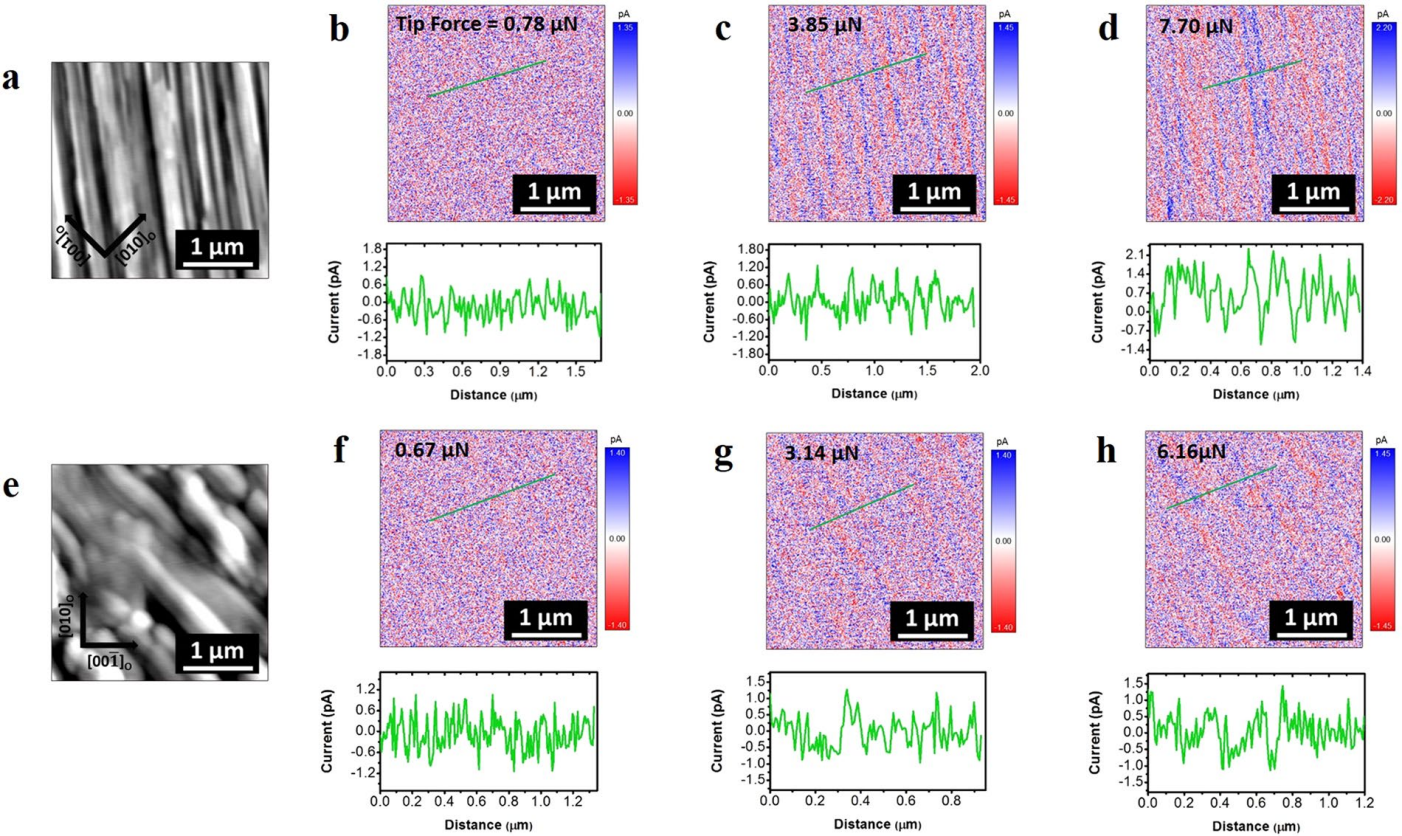
Charge gradient microscopy (CGM) imaging of (**b**–**d**) BFO/NGO and (**f**–**h**) BFO/LSCO/NGO film heterostructures at different applied tip-forces. (**a**) and (**e**) show the AFM topography of the CGM scan areas, respectively. Insets show the section analysis of CGM-current in (**b**–**d**) and (**f**–**h**) images.

We have also performed charge gradient microscopy (CGM) imaging on BFO/NGO and BFO/LSCO/NGO films to confirm the widening of the in-plane polarization variants due to the presence of LSCO interlayer. CGM was used to map the screening charges on ferroelectric domain, which are correlated with the vertical polarization of domains. In CGM imaging of screened ferroelectric surfaces, the grounded conductive CGM-tip scrapes the surface charges, of which flow is measured as the CGM current^24^. CGM measurements were repeated on both films by using different applied tip forces. We observed uniform and high current contrast in the neighboring domains in BFO/NGO film when compared to the BFO/LSCO/NGO film, where in latter one current contrast is suppressed with the expansion in the area with almost no current as shown in Fig. 4. To confirm the reliability of CGM measurements, we performed CGM and PFM scanning together on the same sample area of interest to further confirm the domain structure and correlate the PFM results with CGM current response (Fig. S7). Our CGM results can be explained by the mechanism based on the presence of the external screening charges and scraping these charges due to the mechanical impact of the tip transferred to them as well as repulsive force from other screening charges in the vicinity^24^. In such a case the screening charges of the same polarity will be supplied from the grounded tip to the film surface. Therefore, the current polarity flowing to the tip will be positive for positive domains and negative for negative domains, whereas the CGM current will be negligible for the domains having in-plane direction of polarization^24^.

**Figure 5:**
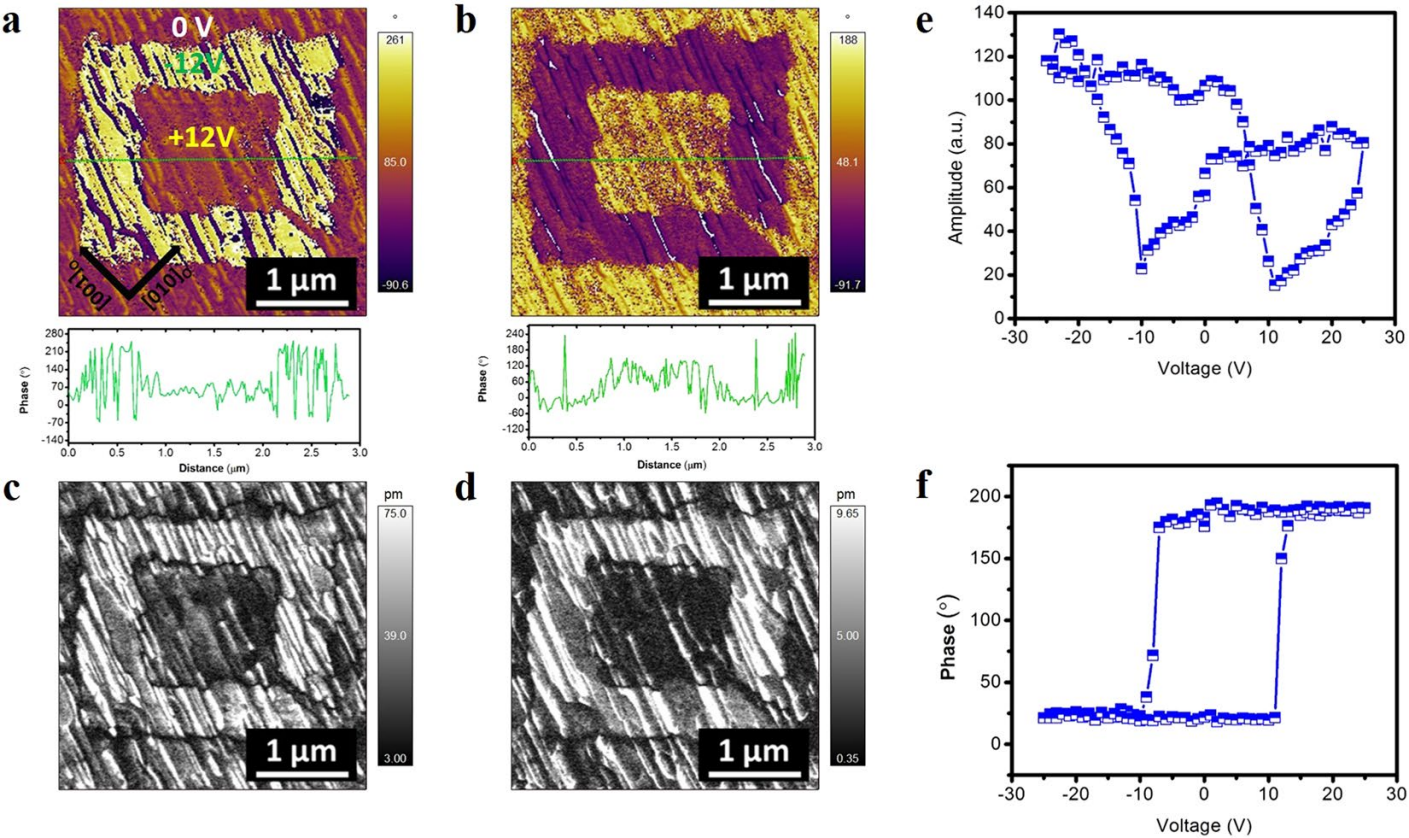
OP-PFM images (using dual AC Resonance tracking (DART) mode) of written domains pattern in BFO films on LSCO/NGO showing (**a**,**b**) phase and (**c**,**d**) amplitude contrasts. Insets show the section analysis of phase change along the dashed line. (**e**) Amplitude and (**f**) phase hysteresis loop.

Furthermore, using PFM switching measurements, we explored the reversibility of stripe domains in BFO/LSCO/NGO by applying dc voltage of ±12 V to the tip. As shown in Fig. 5a–d, the OP phase and amplitude components changed the contrast maintaining the stripe domain morphology. The reason that the stripe domain morphology was maintained during the switching experiment can be found from the PFM results where BFO film with LSCO interlayer contains more in-plane domains, which do not switch to out-of-plane direction but may switch in in-plane direction^25^. Figure 5e,f showed the butterfly amplitude and phase hysteresis loops, respectively, confirming well-defined ferroelectric property of the film.

Doping can induce anisotropy that leads to stripe domain patterns like in the case of lattice mismatch strain induced anisotropy in epitaxially grown undoped BFO thin films with stripe domains^17,21,26^. Stripe domains are attractive as they are related to either 109° or 71° domain walls due to the ferroelastic interactions, which can link ferroelectric polarization with magnetization in BFO thin films. In epitaxial BFO thin films, it has been reported that the A-site substitution with La ions results in straight stripe domains with either 109° or 71° one-dimensional periodic domain walls^21^. However, there has been no report in the literature so far showing that B-site substitution leads to stripe domain structure in BFO thin films.

**Figure 6:**
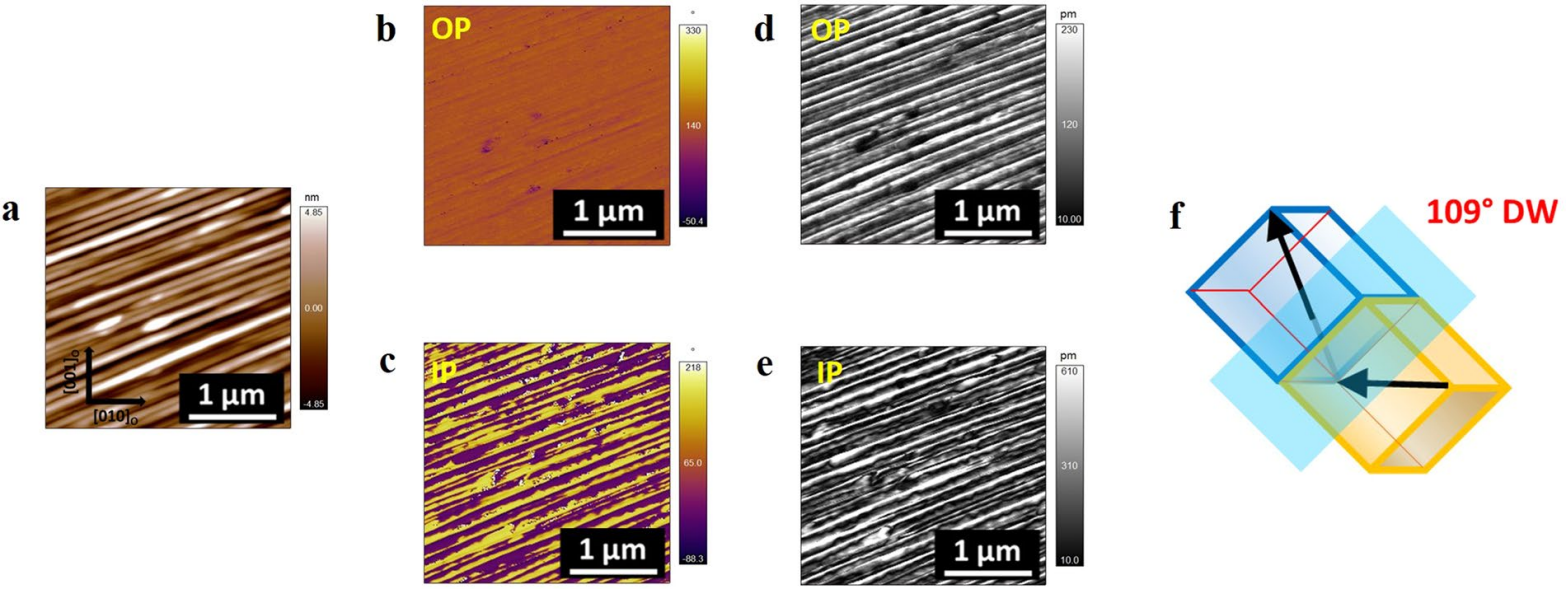
Surface topography (**a**), OP and IP phase (**b**,**c**) and amplitude (**d**,**e**) images of Nb-doped BFO (BFNO) films grown on NGO (100). (**f**) Schematic illustration of the 109° domain structure in BFNO/NGO heterostructure.

Here we substituted Fe (B-Site) with Nb (typically 3% Nb) to investigate the effect of B-site doping on the creation of stripe domains. In addition, the Nb-doped BFO (BFNO) thin films have been reported to show enhanced ferroelectricity^27^. We observed that Nb-doping creates periodic array of long-range stripe nanodomains throughout the film deposited on NGO (100)_o_ substrate. Interestingly, we found that Nb-doping not only creates stripe domains but also changes the domain and domain wall orientations. Analysis of both OP (Fig. 6b,d) and IP (Fig. 6c,e) PFM phase and amplitude images of BFNO/NGO film reveals that the most of the film (scan area) possesses uniform OP phase contrast. This indicates that the film initially has downward polarization. On the other hand, IP phase and amplitude images showed well-aligned periodic stripe nanodomains with phase being alternated between bright and dark contrasts and amplitude being nonuniform across the adjacent domains. We found that domain walls were of the same 109° type (Fig. 6f), whereas the orientation of each domain changed to different directions in BFNO film when compared with BFO film (Fig. S6c).

Different surface morphology of stripe-nanodomains were observed in electrostatically and/or elastically modified interfaces of BFO/NGO and BFO/LSCO/NGO films. The possible reason behind the different surface morphology between two thin film samples with and without LSCO interlayer can be explained in terms of different electrostatic and mechanical boundary conditions at the substrate/film interface. It should be noted that LSCO layer modifies the electrostatic boundary conditions at the bottom interface, where compensation of depolarizing fields by the screening charges could be critical to the stripe morphology in BFO/LSCO/NGO film^6,28^. In case of BFO/NGO heterostructures, the outer surface of the film will be compensated by free charges, since it is exposed to an ambient atmosphere^29^, while the interface between the BFO film and the NGO substrate will be isolated from the free charges as in the case reported for PbTiO_3_/SrTiO_3_ heterostructures^30^. It is known that the equilibrium stripe wave number depends on equilibrium polarization under zero field, dielectric constants, and domain wall energy, which will influence the periodicity as well as the length of the stripe domains. Also, the stripe domains will disappear when the ferroelectric layer can provide charge carriers to compensate the bound charges at the ferroelectric layer/substrate interface, which is similar to the case where LSCO layer is inserted between BFO film and NGO substrate. Indeed, the recent findings reported that insertion of LSMO interlayer between the PTO and the DSO substrate stabilized mono-domain in PTO as it provides free charge compensation to the ferroelectric film at the PTO/LSMO interface whereas the PTO directly grown on the DSO substrate developed stripe domains due to the high in-plane dielectric susceptibility of the strained STO interlayer^28^. Since the stress imposed on BFO by the NGO is more compressive than that by the LSCO, we believe that the boundary conditions imposed on BFO/NGO and BFO/LSCO/NGO result in the similar situation to the PTO/DSO and PTO/LSMO/STO cases. As such, we think that the domain structure is formed as depicted in Fig. 3 to accommodate the asymmetric electrostatic and elastic conditions in vertical direction, which results in the variation of OOP PFM phase and amplitude. CGM results also support that there is an expansion in the in-plane domain variants in the BFO/LSCO/NGO film which resulted in low CGM signal. In case of Nb-doped BFO (BFNO) thin films, we think that doping affects the lattice mismatch between BFO and NGO, which, in turn, alters the effect of in-plane anisotropy imposed by the substrate. Such a doping induced change in anisotropy could be the main reason behind homogeneous long-range stripe morphology and domain pattern in BFNO thin films.

In summary, we have studied the ferroelectric and ferroelastic nanodomains in (110) BiFeO_3_ (BFO) films epitaxially grown on low symmetric orthorhombic (100) NdGaO_3_ (NGO) substrate. We observed the effect of conducting interlayer as well as B-site doping on long range ordering of stripe domains. The combined studies of piezoresponse force microscopy (PFM) and charged gradient microscopy (CGM) revealed 109° periodic vertical domain walls in our thin films. Based on our results, we developed a simple approach to form long-range stripe domains of which domain structure can be tuned by Nb doping. This long-range array of ferroelectric and ferroelastic domains can be useful for optoelectronic devices and ferroelastic templates for strain coupled artificial magnetoelectric heterostructures, which may find their applications in next generation hard disk drives^20,31^.

## Methods

### Film Growth

BFO and Nb-doped BFO films with the thickness of 130 nm were deposited by pulsed laser deposition technique on orthorhombic (*Pbnm*) (100) NGO substrates (with lattice parameters: a = 5.426 Å; b = 5.502 Å; c = 7.706 Å, and lattice parameters for pseudocubic cell: a_p_ = b_p_ = 3.864 Å; c_p_ = 3.853 Å)^32^. The bulk lattice parameter of rhombohedral (*R*$3$*c*) LSCO is; a = 5.419 Å and α_r_ = 60.07°, and a_p_ = 3.836 Å^33^. The laser energy and frequency were 220 mJ and 7 Hz, respectively. The laser energy density was 1.8 J/cm^2^ for target ablation. During film growth, the substrate temperature was kept at 710 °C with an oxygen partial pressure of 80 mTorr. After deposition, the films were cooled down to room temperature at a rate of 10 °C per min with an oxygen partial pressure of 200 mTorr. The LSCO buffer layer (thickness of 100 nm) on NGO was also deposited to fabricate BFO/LSCO/NGO heterostructure. The deposition parameters for LSCO were the same as for BFO films except the oxygen partial pressure, which was 220 mTorr during the LSCO film deposition.

## Characterization

X-ray diffraction (XRD) and Raman spectroscopic studies were carried out to find the orientation and phase formation of BFO films. Raman studies were performed in the backscattering geometry with the excitation line λ = 532 nm by using a Jobin-Yvon T64000 triple spectrometer with grating (1800 grooves mm^−1^). The epitaxial growth of films was confirmed by x-ray diffraction (XRD) scans using Rigaku SmartLab X-ray diffractometer with Cu *K*α radiation.

The surface topography, piezoelectric properties, and domain patterns of these heterostructures were investigated using a commercial atomic force microscope (AFM) (Asylum Research, MFP-3D). Piezoresponse force microscopy (PFM) was performed at ac modulation voltage of 1 V_pp_ (peak to peak) and scan frequency of 1 Hz. The vertical and lateral PFM images were obtained with drive frequencies at 325.45 and 689.62 kHz, respectively, near the contact resonance for vertical and torsional motions of PFM cantilevers. In BFO/LSCO/NGO film, an ac modulation voltage of 1 V was applied to the Pt-coated Si tip (PPP EFM tips, Nanosensors, 2.6–3.0 N/m) while grounding the bottom LSCO electrode. To obtain line profiles across the domain boundaries, an arbitrary line was chosen nearly perpendicular to the domain wall.The vertical piezoresponse hysteresis loops were measured by dual ac resonance tracking PFM (DART-PFM) mode at three different arbitrary points for four times at each position.

We performed our CGM imaging using grounded Pt-wire tips (RMN 25Pt300B, 18 N/m, Rocky Mountain Nanotechnology, LLC) on BFO films. Firstly, we measured the offset voltage and current using highly ordered pyrolytic graphite (HOPG) sample. Then we collected the current through the grounded AFM tip attached to the cantilever holder (ORCA, gain of 5 × 10^8^ volts/amp (~1 pA to 20 nA), Asylum Research) while removing the offset voltage (~−83 mV) and the offset current (~50 pA). The scan frequency and applied force to the tip were varied from 10 to 30 Hz, and 0.78 to 7.77 μN, respectively. All the scanning images consisted of 256 × 256 pixels. The cross-sectional TEM specimens were prepared by focused ion beam (FIB), and then examined using FEI Tecnai F20ST TEM/STEM system with a field emission 200 kV S/TEM.

## Electronic supplementary material

Supplementary Information
