# Author Correction: Long-range Stripe Nanodomains in Epitaxial (110) BiFeO_3_ Thin Films on (100) NdGaO_3_ Substrate

**DOI:** 10.1038/s41598-018-21747-6

**Published:** 2018-03-01

**Authors:** Yogesh Sharma, Radhe Agarwal, Charudatta Phatak, Bumsoo Kim, Seokwoo Jeon, Ram S. Katiyar, Seungbum Hong

**Affiliations:** 1 Materials Science Division, Argonne National Laboratory, Lemont, IL 60439 USA; 2 Department of Physics and Institute for Functional Nanomaterials, University of Puerto Rico, San Juan, PR 00936 USA; 3 Department of Materials Science and Engineering, KAIST, Daejeon, 34141 Korea; 4 Present Address: Materials Science and Technology Division, Oak Ridge National Laboratory, Oak Ridge, TN 37831 USA

Correction to: *Scientific Reports* 10.1038/s41598-017-05055-z, published online 07 July 2017

The Acknowledgements section in this Article is incomplete.

“Work at Argonne (Y.S., C.D., B.K., S.H., PFM, TEM and CGM imaging experiments, data analysis and contribution to manuscript writing) was supported by the US Department of Energy, Office of Science, Basic Energy Sciences, Materials Sciences and Engineering Division. Use of the Center for Nanoscale Materials was supported by the US Department of Energy, Office of Science, Office of Basic Energy Sciences, under contract no. DE-AC02-06CH11357. Y.S. acknowledges the NSF (Grant #1002410) for supporting the guest graduate studentship at Argonne National Laboratory. R. A. acknowledges the NSF (Grant #1002410) for graduate fellowship at Univ. Puerto-Rico. B.K. acknowledges the Brain Korea program (National Research Foundation, Korea) for the guest graduate studentship at Argonne National Laboratory.”

should read:

“C.P. and S.H., were supported by the US Department of Energy, Office of Science, Basic Energy Sciences, Materials Sciences and Engineering Division. Use of the Center for Nanoscale Materials was supported by the US Department of Energy, Office of Science, Office of Basic Energy Sciences, under contract no. DE-AC02-06CH11357. Y.S. acknowledges the NSF (Grant #1002410) for supporting a guest graduate studentship at Argonne National Laboratory. R. A. acknowledges the NSF (Grant #1002410) for a graduate fellowship at Univ. Puerto-Rico. B. K. acknowledges the Brain Korea program (National Research Foundation of Korea) for supporting a guest graduate studentship at Argonne National Laboratory.”

