# Supplementary material for: Long-range Stripe Nanodomains in Epitaxial (110) BiFeO3 Thin Films on (100) NdGaO3 Substrate
Source: Sci Rep. 2017 Jul 7;7:4857. doi: 10.1038/s41598-017-05055-z (PMC5501854; doi:10.1038/s41598-017-05055-z)
Supplement: Supplementary file 1 — Supplementary Information [file 41598_2017_5055_MOESM1_ESM.pdf]

Supplementary Information

Long-range Stripe Nanodomains in Epitaxial (110) $BiFeO_3$ Thin Films on (100) $NdGaO_3$ Substrate

Yogesh Sharma,[a, b] Radhe Agarwal,[b] Charudatta Phatak,[a] Bumsoo Kim,[a,c] Seokwoo Jeon,[c] Ram S. Katiyar,[b] and Seungbum Hong[a,c,1]

[a] *Materials Science Division, Argonne National Laboratory, Lemont, IL 60439, USA.*

[b] *Department of Physics and Institute for Functional Nanomaterials, University of Puerto Rico, San Juan, PR-00936-8377, USA.*

[c] *Department of Materials Science and Engineering, KAIST, Daejeon 305-701, Korea.*

[1] Corresponding author: seungbum@kaist.ac.kr

XRD peak shift

A comparison of the (110) peak position of BFO, BFO/LSCO, and BFNO films was presented in Figure S1 a. We observed that 2θ value of (110) peak shifts towards lower angle side when compared to the bulk value in all films indicating that our films have in-plane compressive strain. However, in case of BFO film grown on LSCO buffered NGO substrate this shift is not much pronounced because of suppression of in-plane anisotropic strain imposed by NGO substrate. We have tabulated the 2θ value corresponding to (110) peak for all the samples in Figure S1 b. XRD pattern of BFNO sample is presented in Figure S1 c.

a

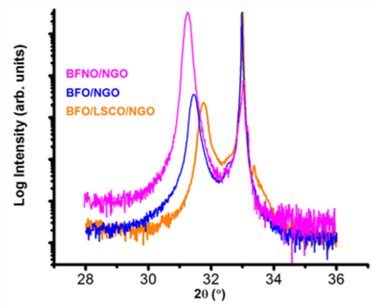

b

Thin film on NGO (100) substrate	2θ (degree)	
	Bulk	Film
LSCO (110)	33.42	33.59
BFO (110)	32.07	31.48
BFO/LSCO (110)	---	31.86
BFNO (110)	---	31.29

c

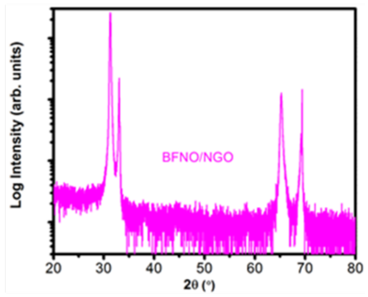

Figure S1 a. (110) peak position of BFO, BFO/LSCO, and BFNO films grown on NGO (100), b. table for the 2θ value of (110) for all the samples, c. XRD pattern of BFNO film.

Puckering angle

We calculated the puckering angle using the line profile data of AFM topography image. We selected different peaks from line-profile data and calculated the puckering angle using simple trigonometry equations. For example, we selected a peak from line-profile, and after finding the height and width of the peak, we use simple cosine formula to calculate the puckering angle (Figure

S2). We calculated the average puckering angles from 4 peaks, which are 174° (for BFO/NGO), 174.8° (for BFO/LSCO/NGO), and 173.7° (for BFNO/NGO), respectively.

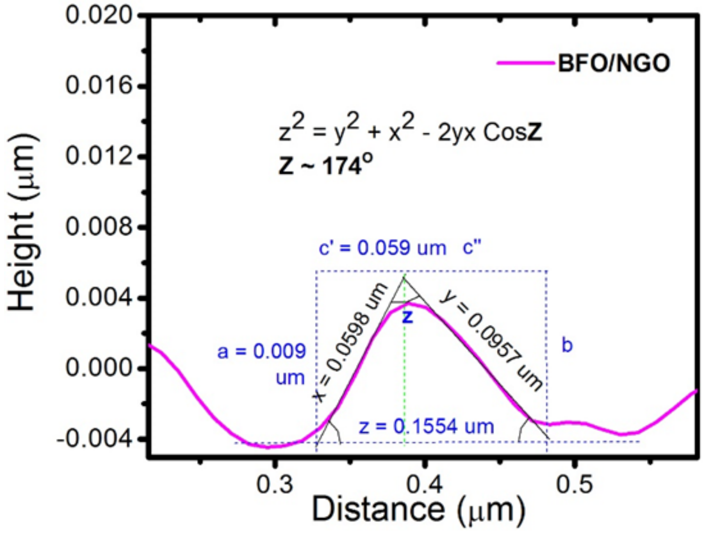

Figure S2 Puckering angle calculation using the line profile data of AFM topography image for BFO/NGO film.

Angle resolved PFM measurements

The mixed domain formation in BFO/NGO films were further confirmed by angle resolved PFM measurements, whereby the sample was rotated by 45° increments around the normal to the film surface and OP and IP PFM phase and amplitude signals were collected at 0°, 45°, and 90°, respectively, as shown in Fig. S3. The correlations between IP phase and amplitude responses at 90° rotation of sample with the OP phase and amplitude responses, can confirm the presence of 109° stripe domain patterns in BFO/NGO film.

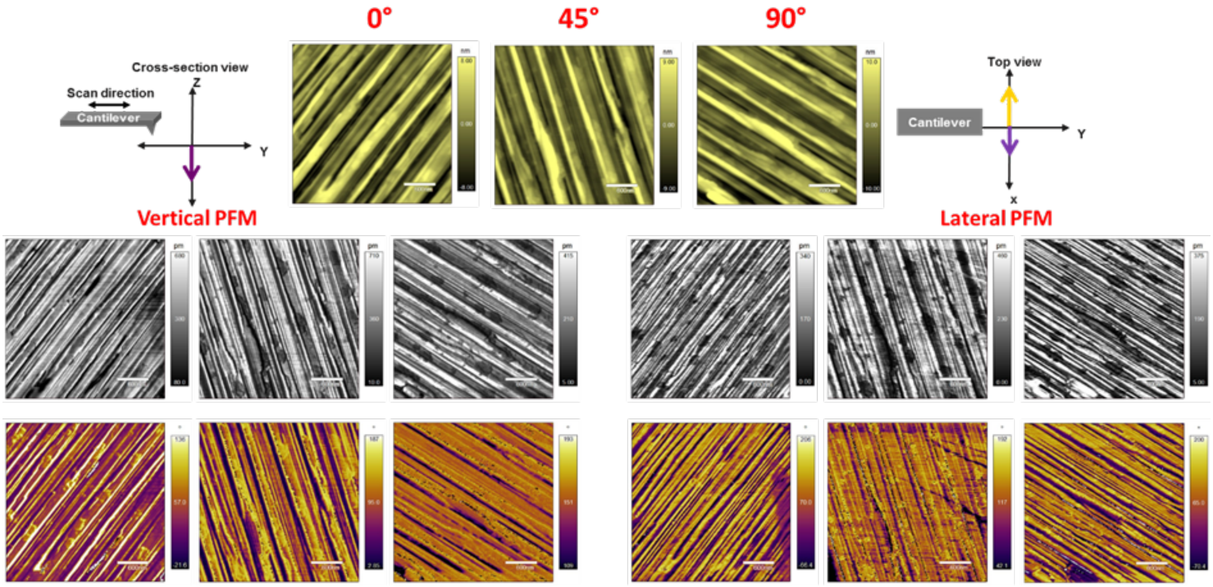

Figure S3 Angle resolved PFM (AR-PFM) images of BFO/NGO heterostructure thin films at three different angles of the rotation of the sample. Scale bar is 600 nm.

<u>Cross-sectional TEM and SEM measurements:</u>

Cross-sectional transmission electron microscopy (TEM) studies were performed on BFO/NGO sample. TEM specimen sample were prepared using focused ion beam (FIB) lift-out method. Figure S4 (a-c) shows the dark field TEM image of BFO/NGO heterostructure taken under beam condition with g = $[200]_O$ direction. The thickness of the film is estimated of ~ 130 nm. The appearance of triangular nanodomain indicates the presence of ferroelectric/ferroelastic domain walls in our BFO films.[1,2] High resolution-TEM (HRTEM) image also confirms the presence of domain walls in (110)-BFO film. In Figure S4 (d), cross-sectional SEM image of BFO/LSCO/NGO film was provided. The SEM image confirmed that the thickness of LSCO conducting layer is around 100 nm.

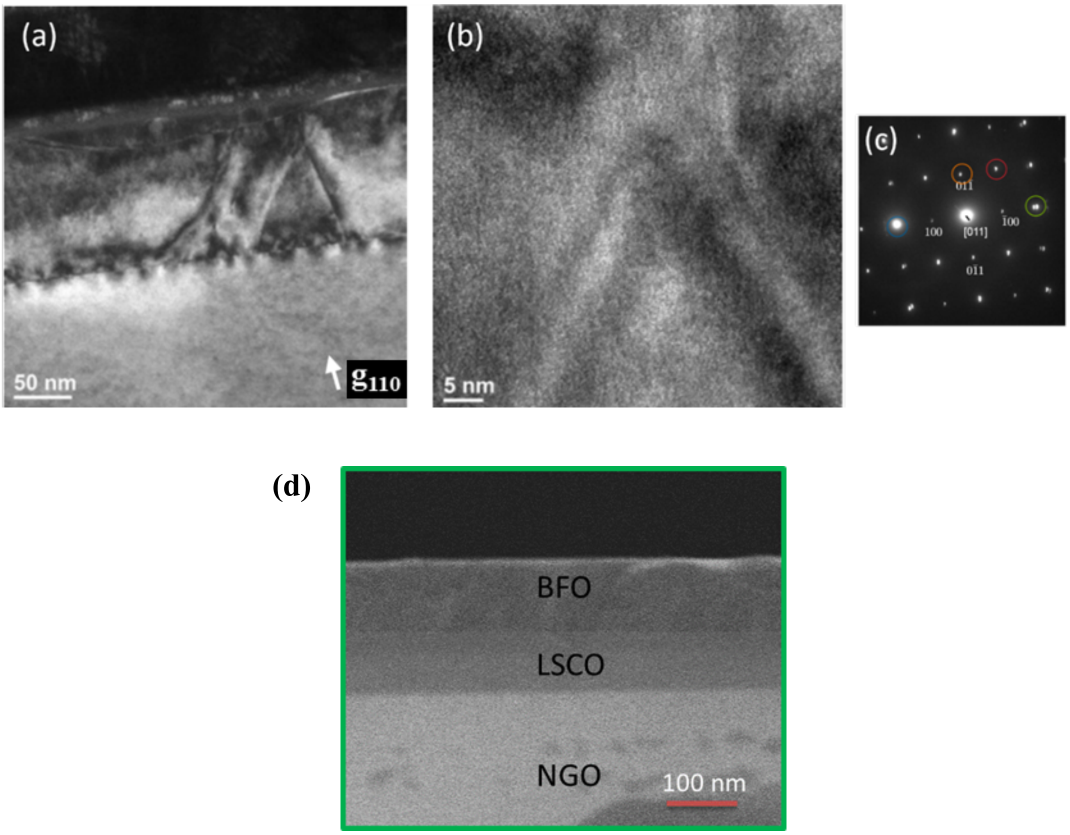

Figure S4 (a) Dark field TEM cross-sectional image, (b) HRTEM image of the domain wall features, and (c) SAED pattern from the BFO/NGO heterostructure. (d) SEM cross-sectional image of BFO/LSCO/NGO heterostructure.

Comparative analysis of PFM phase and amplitude images of BFO thin films with the possible combination of domain variants

Schematics of possible combinations of polarization variants with 180°, 109°and 71° domain walls are presented in Fig. S5, considering out-of-plane (OP) and in-plane (IP) motions of the tip.

180 Deg.

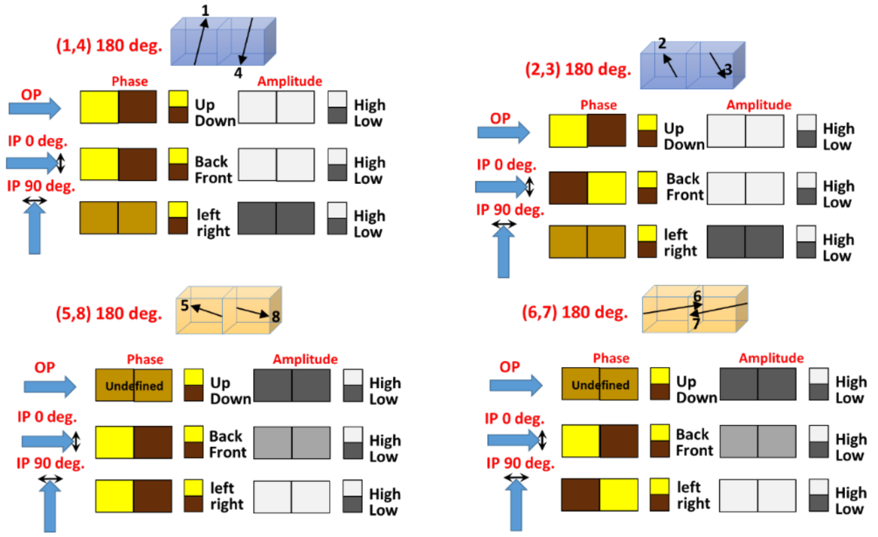

109 Deg.

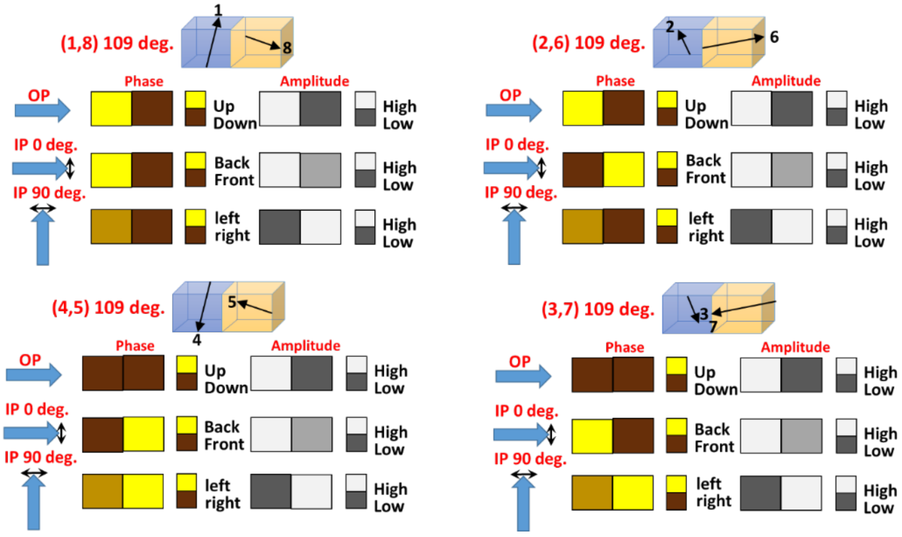

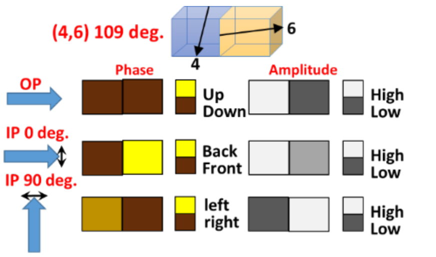
(4,6) 109 deg.
4
6
Phase
Amplitude
OP
IP 0 deg.
IP 90 deg.
Up
Down
Back
Front
left
right
High
Low
High
Low
High
Low

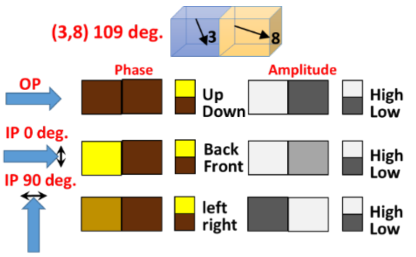
(3,8) 109 deg.
3
8
Phase
Amplitude
OP
IP 0 deg.
IP 90 deg.
Up
Down
Back
Front
left
right
High
Low
High
Low
High
Low

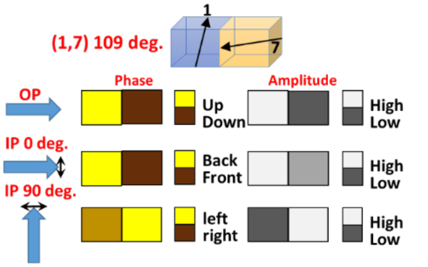
(1,7) 109 deg.
1
7
Phase
Amplitude
OP
IP 0 deg.
IP 90 deg.
Up
Down
Back
Front
left
right
High
Low
High
Low
High
Low

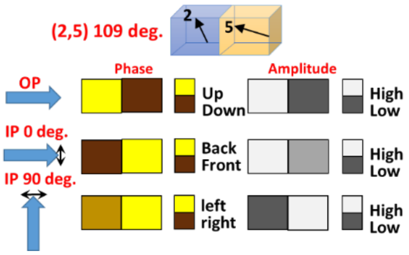
(2,5) 109 deg.
2
5
Phase
Amplitude
OP
IP 0 deg.
IP 90 deg.
Up
Down
Back
Front
left
right
High
Low
High
Low
High
Low

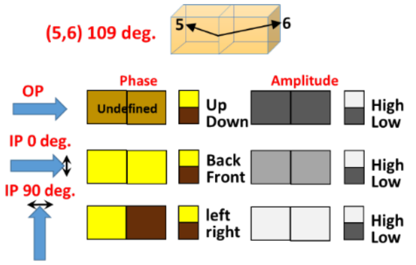
(5,6) 109 deg.
5
6
Phase
Amplitude
OP
IP 0 deg.
IP 90 deg.
Undefined
Up
Down
Back
Front
left
right
High
Low
High
Low
High
Low

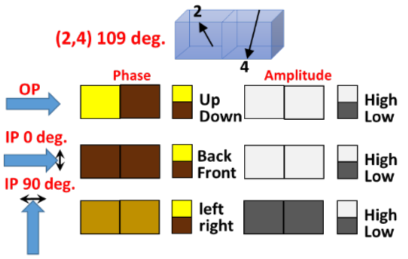
(2,4) 109 deg.
2
4
Phase
Amplitude
OP
IP 0 deg.
IP 90 deg.
Up
Down
Back
Front
left
right
High
Low
High
Low
High
Low

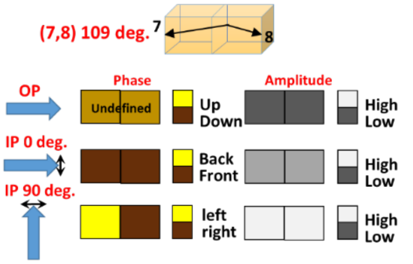
(7,8) 109 deg.
7
8
Phase
Amplitude
OP
IP 0 deg.
IP 90 deg.
Undefined
Up
Down
Back
Front
left
right
High
Low
High
Low
High
Low

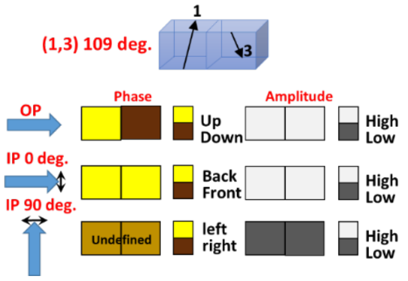
(1,3) 109 deg.
1
3
Phase
Amplitude
OP
IP 0 deg.
IP 90 deg.
Up
Down
Back
Front
Undefined
left
right
High
Low
High
Low
High
Low

71 Deg.

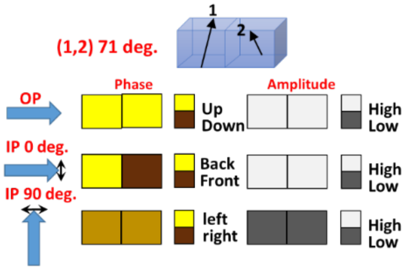
(1,2) 71 deg.
1
2
Phase
Amplitude
OP
IP 0 deg.
IP 90 deg.
Up
Down
Back
Front
left
right
High
Low
High
Low
High
Low

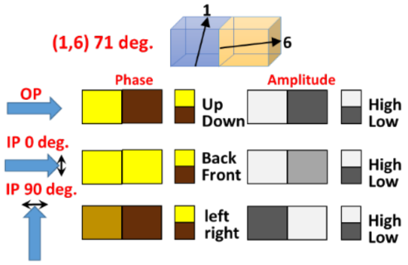
(1,6) 71 deg.
1
6
Phase
Amplitude
OP
IP 0 deg.
IP 90 deg.
Up
Down
Back
Front
left
right
High
Low
High
Low
High
Low

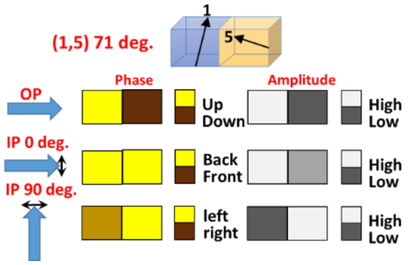
(1,5) 71 deg.
1
5
Phase
Amplitude
OP
IP 0 deg.
IP 90 deg.
Up
Down
Back
Front
left
right
High
Low
High
Low
High
Low

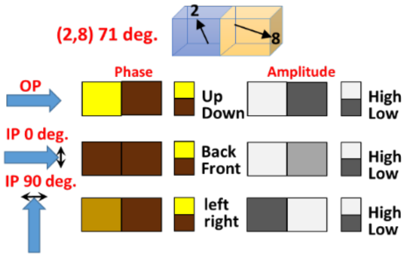
(2,8) 71 deg.
2
8
Phase
Amplitude
OP
IP 0 deg.
IP 90 deg.
Up
Down
Back
Front
left
right
High
Low
High
Low
High
Low

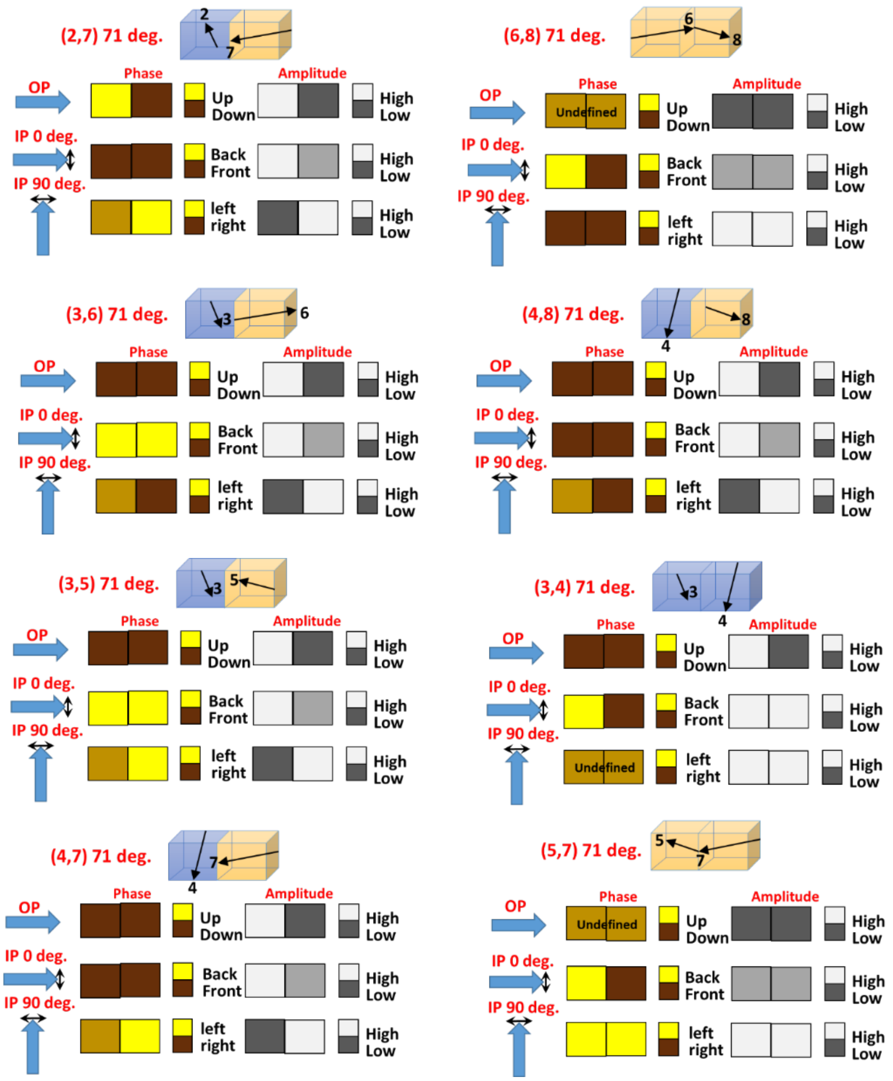

Figure S5 Schematic representation of possible bi-domain variants with 180°, 109° and 71° domain walls and their respective OP and IP phase and amplitude contrast based on measurement conditions.

The observed domain and domain wall orientations were determined by comparing PFM phase and amplitude response with the schematic representations of different polarization variants corresponding to180°, 109° and 71° domain walls orientations. Figure S6 (a-c) shows the selected area OP and IP phase and amplitude contrast and their suitable match with the possible domain bi-variants from Fig. S5. Based on this analysis, the schematics of domain and domain wall

orientations were also presented for BFO/NGO (a), BFO/LSCO/NGO (b), and BFNO/NGO (c) films.

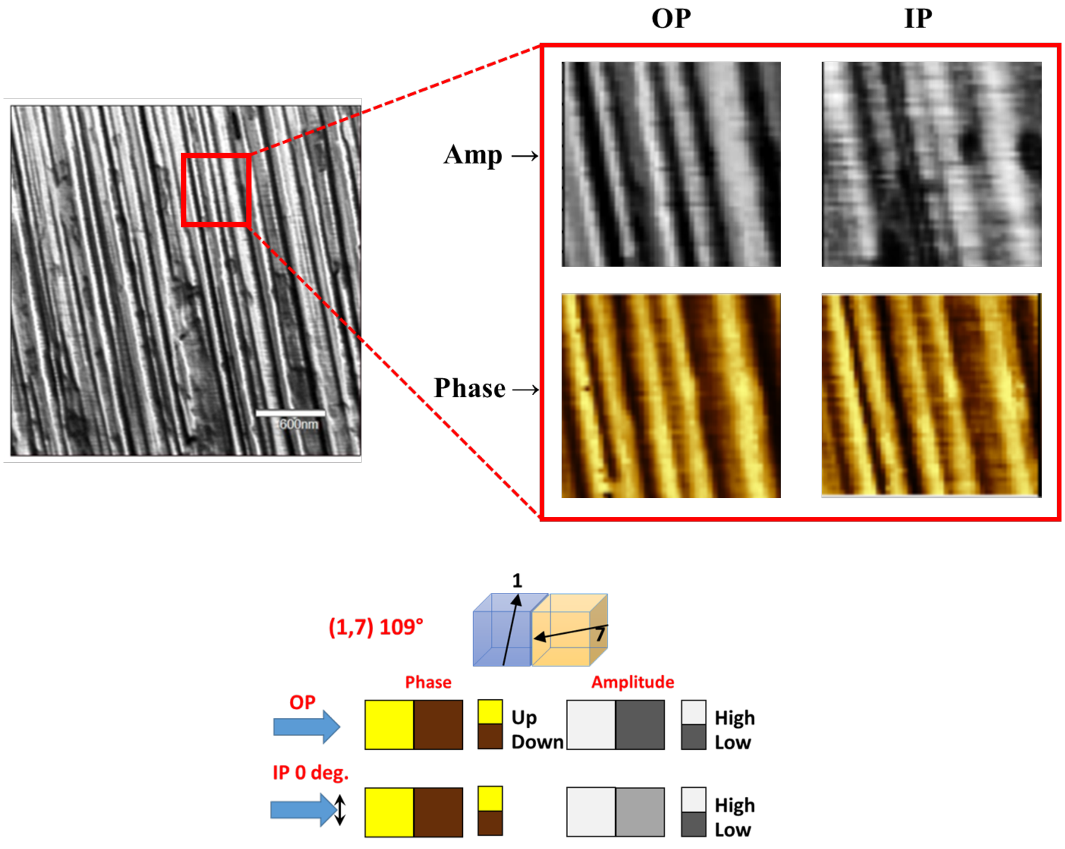

(a)

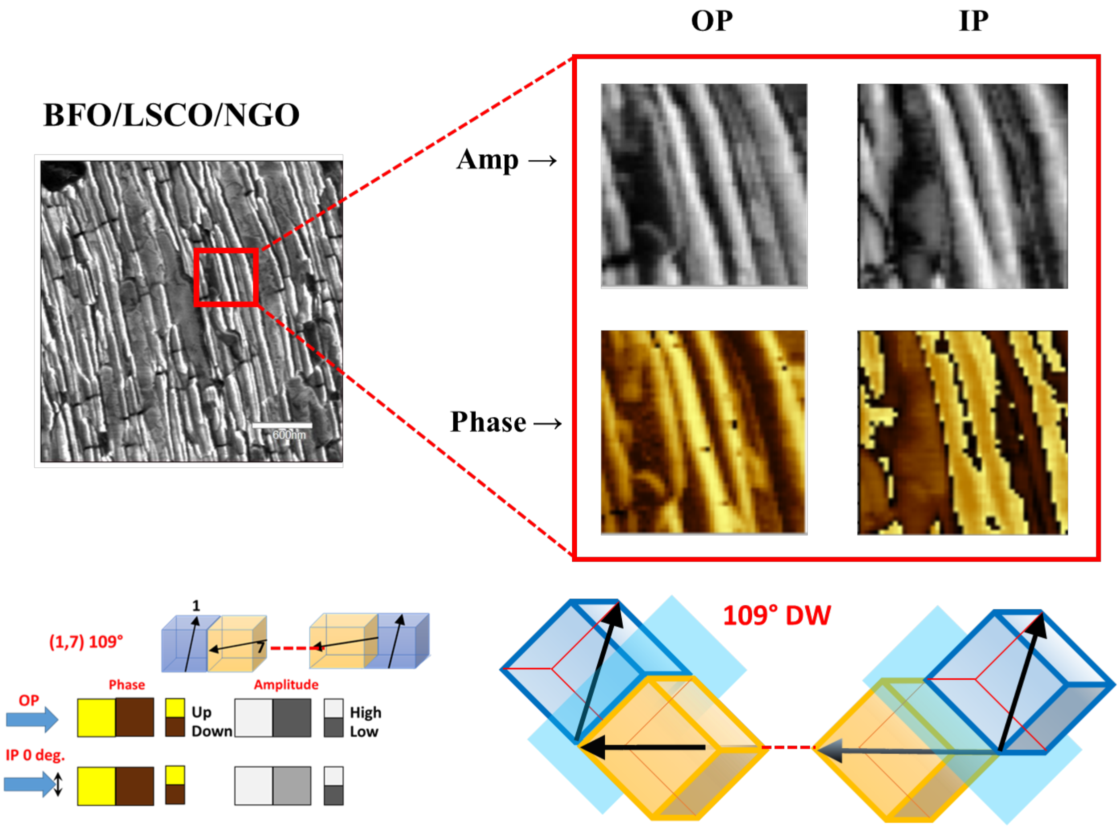
OP
IP
BFO/LSCO/NGO
Amp →
Phase →
600nm
(1,7) 109°
1
7
Phase
Amplitude
OP
Up
Down
High
Low
IP 0 deg.
109° DW

(b)

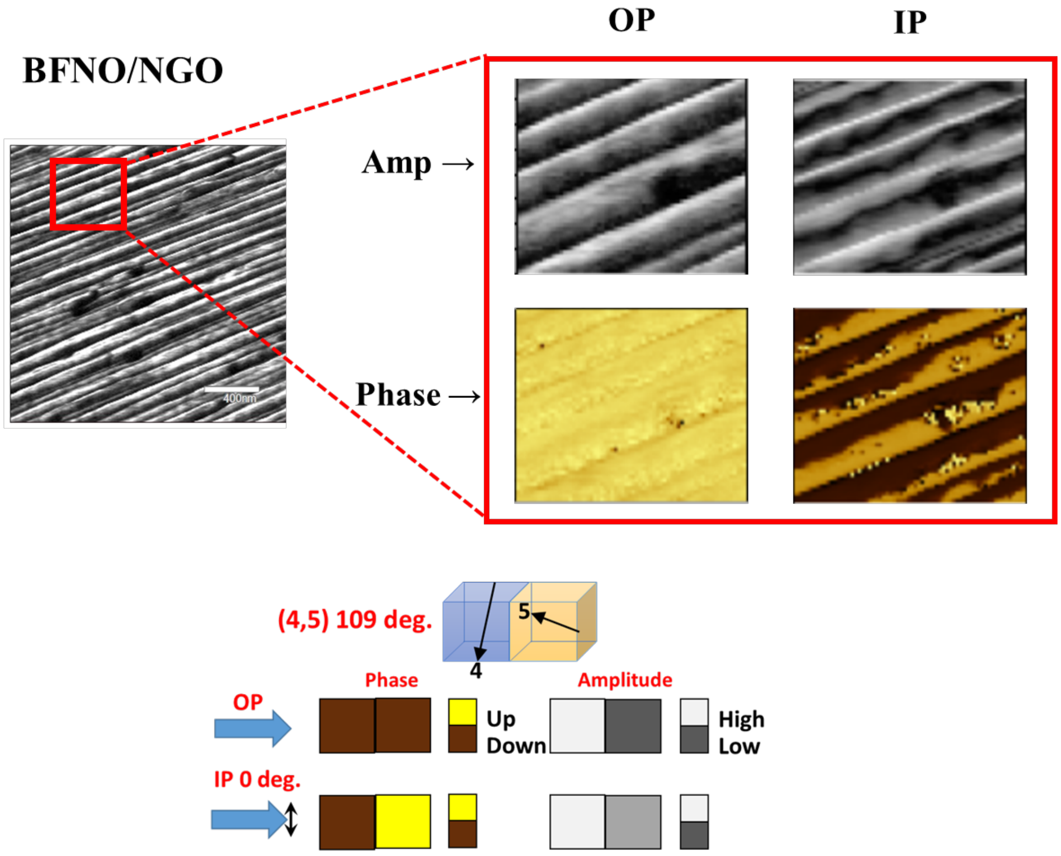

(c)

Figure S6 PFM phase and amplitude images of (a) BFO/NGO, (b) BFO/LSCO/NGO, and (c) BFNO/NGO films with the schematics of possible domain variants having 109° domain wall. Scale bar is 600 nm for (a) and (b), and 400 nm for (c).

<u>CGM and PFM scanning together on the same sample area</u>

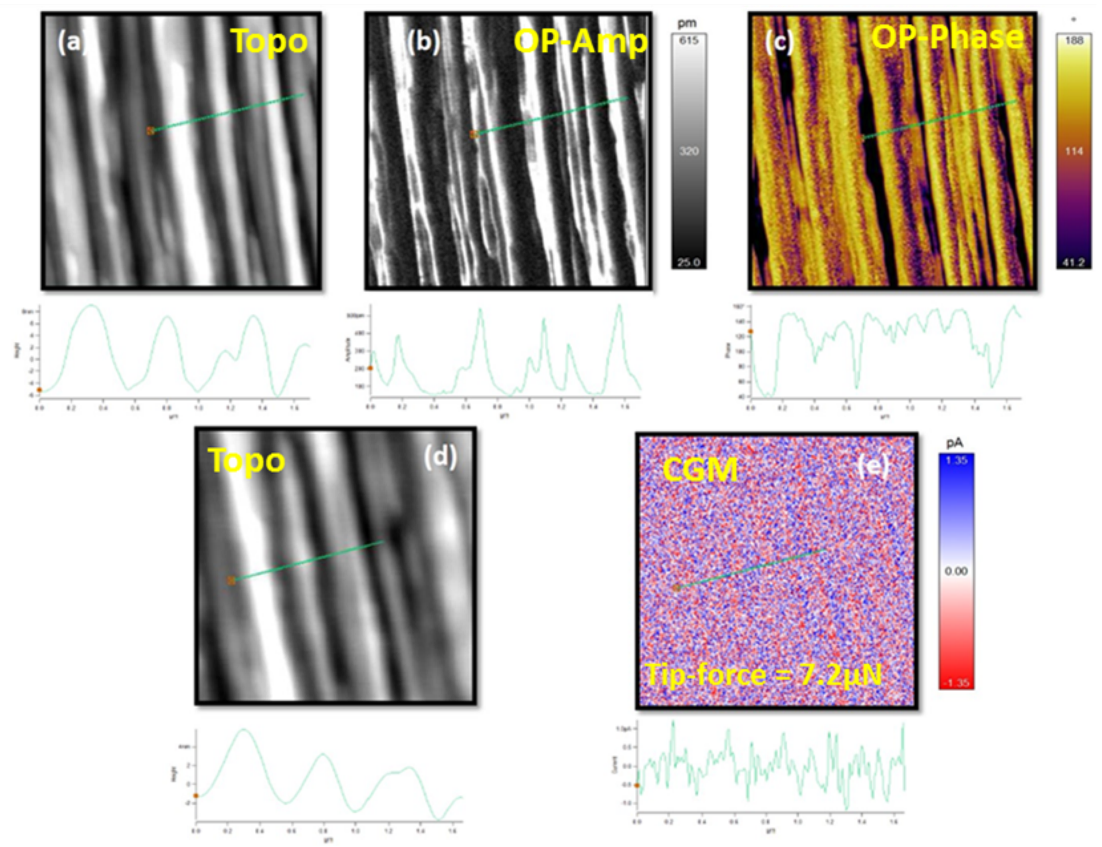

Figure S7 PFM and CGM scanning on the same area of the sample BFO/NGO. (a) and (d) represent the topography before and after the CGM scan, respectively. (b) and (c) are the OP-amplitude and phase images. (e) CGM image with the current contrast in the same sample area where PFM measurements were performed. Insets show the section analysis of line profile of height, PFM-phase and amplitude, and CGM-current. Scale bar 500 nm.
